# Supplementary material for: An immune receptor complex evolved in soybean to perceive a polymorphic bacterial flagellin
Source: Nat Commun. 2020 Jul 28;11:3763. doi: 10.1038/s41467-020-17573-y (PMC7387336; doi:10.1038/s41467-020-17573-y)
Supplement: Supplementary file 2 — Supplementary Information [file 41467_2020_17573_MOESM2_ESM.pdf]

Supplementary Information

An immune receptor complex evolved in soybean to perceive a polymorphic bacterial flagellin

Wei et al.

Includes:

- **Supplementary Figures 1-15.**
- **Supplementary Tables 1 and 2.**

Supplementary Figure 1

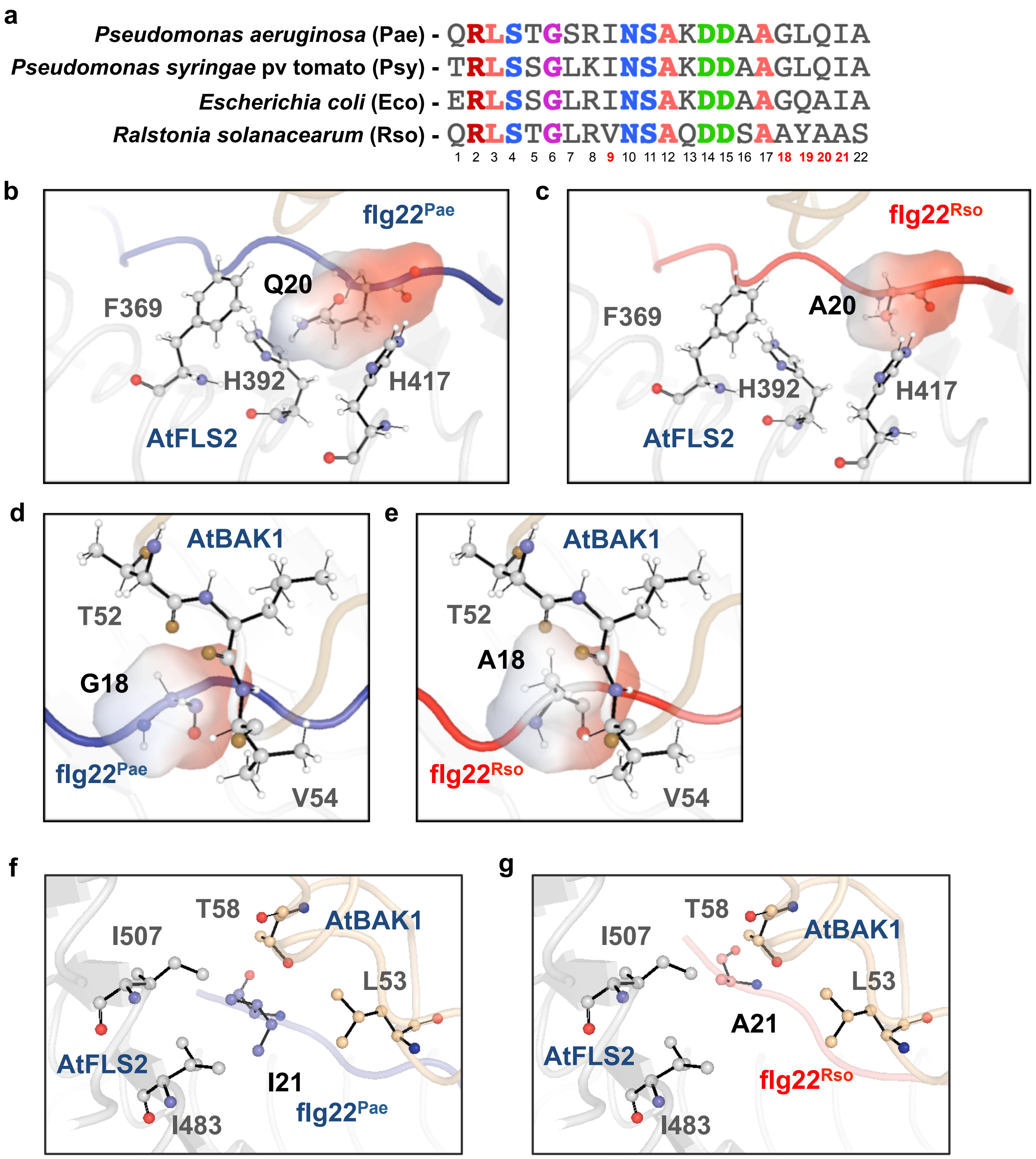

Supplementary Figure 1. Analysis of polymorphisms in flg22^{Rso}.

a. Sequence alignment of the flg22 peptide from *Ralstonia solanacearum* GMI1000 and other flg22 peptides perceived by Arabidopsis thaliana. Note that the flg22 peptide from different *Pseudomonas* species, including *P. aeruginosa* (Pae) and the plant pathogen *P. syringae* (Psy) display high similarity, showing identical sequence in the residues 9-22. **b.** Interactions between flg22^{Pae} and AtFLS2 around Q20. **c.** Change in structure around this position upon mutation to the A20 present in flg22^{Rso}. It is possible that the presence of the amide group at the complex interface facilitates the formation of amino-aromatic interactions between Q20 and residues in the FLS2 interface, which are lost when an alanine is present in this position. **d.** Interactions between flg22^{Pae} and AtBAK1 around G18. **e.** Change in structure around this position upon mutation to the A18 present in flg22^{Rso}. Glycine is a very unique amino acid in the sense that it contains a hydrogen as its side chain (rather than a carbon as is the case in all other amino acids). This means that there is much more conformational flexibility in glycine, providing glycine-containing regions of the peptide with the ability to make tight turns and kinks. Glycine is also unique in the sense that it basically has no side chain and can therefore allow for easily accessible backbone-to-backbone interactions. It appears this might be the case in this situation, where a simple substitution to alanine interrupts contacts to the backbone of the T52 of BAK1. **f and g.** Different angle of the location represented in Figure 1c and 1d, respectively showing the modeled interactions around I21 and the alteration in this structure upon mutation of this residue to the A21.

Supplementary Figure 2

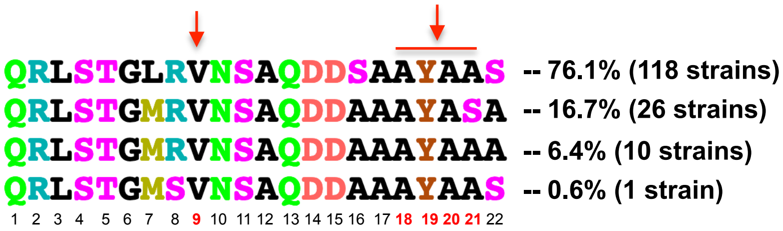

Supplementary Figure 2. Alignment of the flg22 sequences present in all the sequenced *R. solanacearum* strains.
Flg22 sequences were extracted from 155 sequenced *R. solanacearum* strains and aligned to identify amino acid polymorphisms. The amino acids highlighted in our study (key for evasion of perception by FLS2/BAK1) are indicated with arrows. The name of the sequenced strains and the metadata associated to the genome sequences has been previously published (Sabbagh et al, 2019).

Supplementary Figure 3

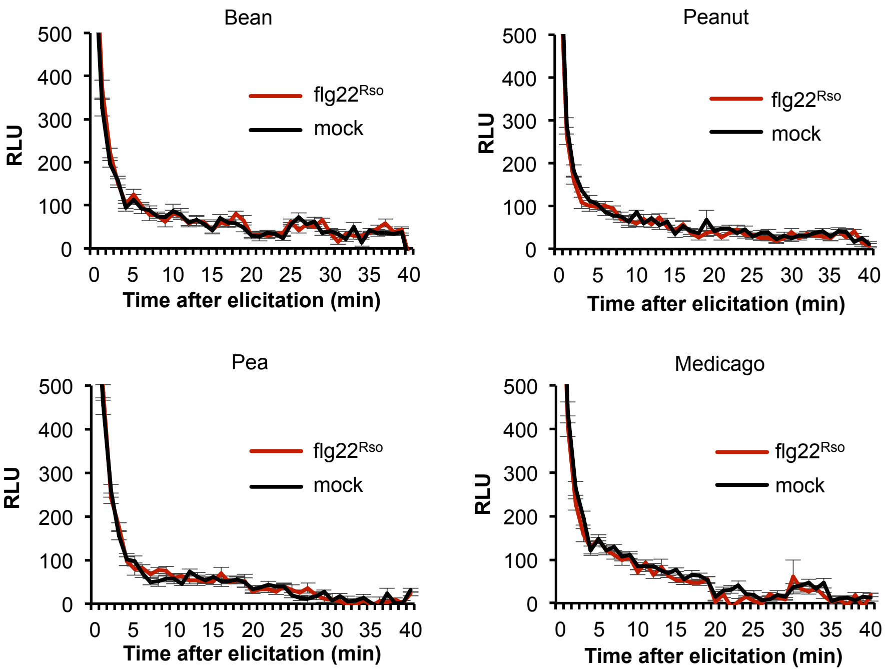

Supplementary Figure 3. Dynamics of ROS burst triggered by flg22^{Rso} in different plant species. Dynamics of ROS burst corresponding to the assays in the Figure 2a, triggered by flg22^{Rso} (100 nM) or a mock (water) treatment in leaf discs from the indicated plant species, measured in a luminol-based assay, and represented as accumulated relative luminescence units (RLU) (mean ± SEM, n=8). The measurement was performed from 1 to 40 min after treatment. The scale used is the same as in the figure 2b, to allow for a comparison with the ROS dynamics in soybean. These experiments were performed 3 times with similar results.

Supplementary Figure 4

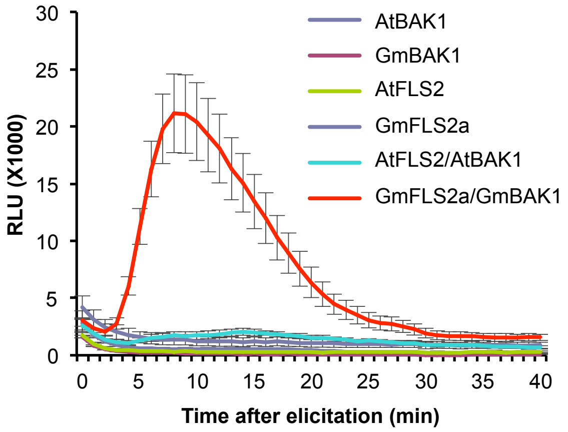

Supplementary Figure 4. The overexpression of Arabidopsis PRRs does not confer responsiveness to flg22^{Rso}. Dynamics of ROS burst triggered by flg22^{Rso} (100 nM) in *N. benthamiana* leaf tissues expressing the indicated PRRs , from a *35S* promoter. ROS was measured in a luminol-based assay, and represented as accumulated relative luminescence units (RLU) (mean ± SEM, n=8). The measurement was performed from 1 to 40 min after treatment. This experiment was performed 3 times with similar results.

Supplementary Figure 5

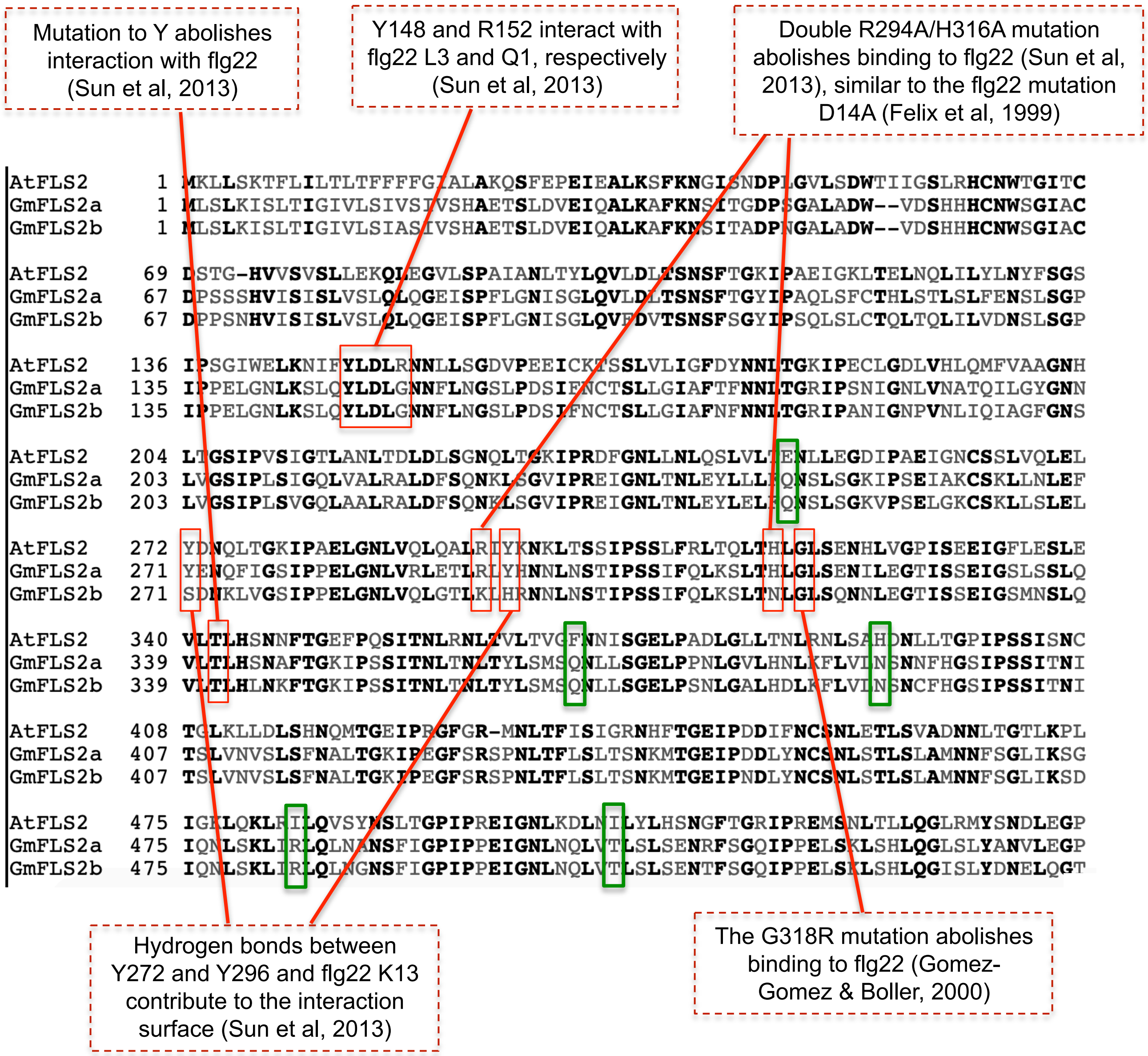

Supplementary Figure 5. Annotated alignment of the extracellular domains of GmFLS2a, GmFLS2b, and AtFLS2.
Alignment of the residues 1-542 (within the extracellular domain) of AtFLS2, GmFLS2a, and GmFLS2b. The annotation in red highlights several residues shown to be important for binding to flg22 (Felix et al, 1999; Gomez-Gomez & Boller, 2000; Sun et al, 2013). Green boxes indicate the residues selected for analysis in Figure 3.

Supplementary Figure 6

Residues that interact with FLS2

Residues 52-54 make an inner-curved loop that accommodates flg22 G18

```
AtBAK1    1 MERR---LMIPCFFWLILVLDLVLRVSGNAEGDALSALKNSLADPNKVLQSWDATLVTPCTWFHVTCNSD
GmBAK1    1 MERVTSSFMGSFFFWAILVLDLVLKASGNQEGDALNALKSNLQDPNNVLQSWDATLVNPCTWFHVTCNSD

AtBAK1   68 NSVTRVDLGNANLSGQLVMQLGQLPNLQYLELYSNNITGTIPEQLGNLTELVSLDLYLNNLSGPIPSTLG
GmBAK1   71 NSVTRVDLGNADLSGQLVSQLGQLTNLQYLELYSNKITGKIPDELGNLTNLVSLDLYLNTLNGPIPTTLG

AtBAK1  138 RLKKLRFLRLNNNSLSGEIPRSLTAVLTLQVLDLSNNPLTGDIPVNGSFSLFTPISFANTKLTPLPASPP
GmBAK1  141 KLAKLRFLRLNNNSLTGGIPISLTNVSSLQVLDLSNNHLKGEIPVNGSFSLFTPISYQNNLGLIQPKYTP

AtBAK1  208 PPISPTPPSPAGSNRITGAIAGGVAAGAALLFAVPAIALAWWRRKKPQDHFFDVPAEEDPEVHLGQLKRF
GmBAK1  211 SPVSPTPPPASSGNSNTGAIAGGVAAGAALLFAAPAIALAYWRRRKPQDHFFDVPAEEDPEVHLGQLKRF
```

Supplementary Figure 6. Annotated alignment of the extracellular domains of GmBAK1 and AtBAK1.
Alignment of the residues 1-277 (within the extracellular domain) of AtBAK1 and GmBAK1. Residues shown to mediate interaction with FLS2 are marked with blue boxes. The annotation highlights additional residues important for binding to flg22 (Sun et al, 2013). A green box indicates the N58T mutation analyzed in the Supplementary Figure 11.

Supplementary Figure 7

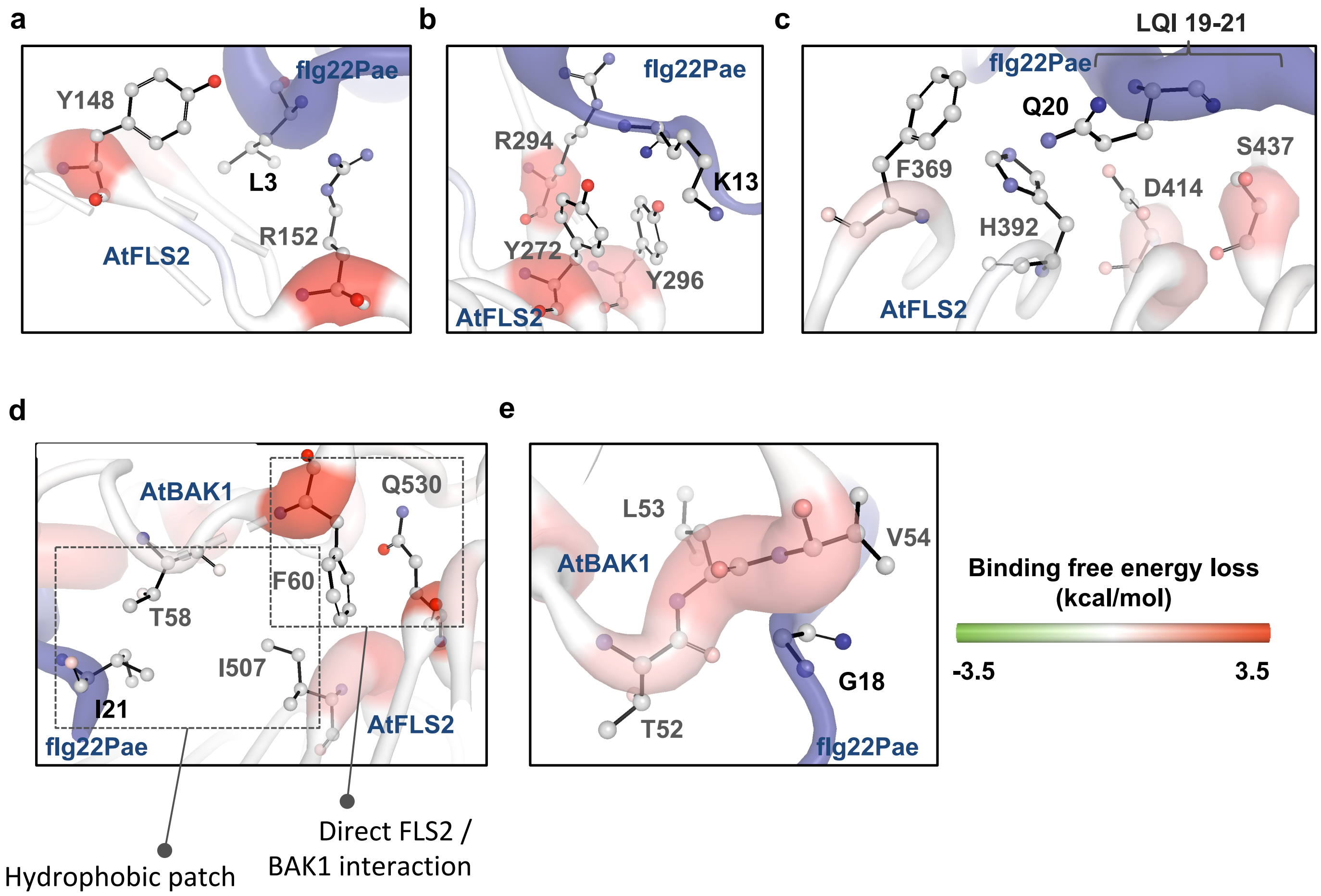

Supplementary Figure 7. Illustration of the hotspots in the FLS2/flg22/BAK1 complex where polymorphisms within the PRRs may impact flg22 perception.
Alanine Scanning of the all interface residues in the complex was performed using the Robetta server. The procedure identifies residues that are involved in the protein–protein interface, and uses a simple free energy function to calculate the changes in the binding free energy upon single substitutions of each side chain to alanine. The two presumed stages of the recognition process were analyzed separately: first the binding of flg22 to FLS2 (panels **a-c**), then the binding of BAK1 to the complex formed by the other two (panels **d and e**). A color code was used to represent binding free energy loss, and the flg22 peptide is indicated in purple to differentiate it from PRRs.

Supplementary Figure 8

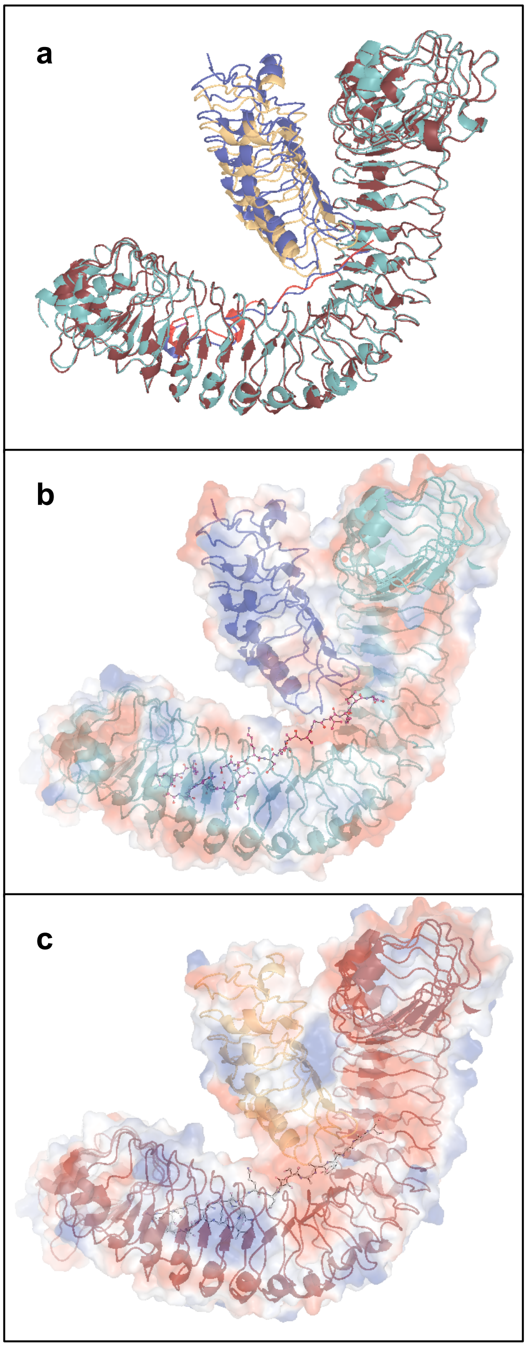

Supplementary Figure 8. Homology-based modeling of the structure of the extracellular domains of GmFLS2b and GmBAK1 bound to flg22^{Rso}, using the AtFLS2/AtBAK1/flg22^{Pae} structure as template.
a. Superimposed structures of the two ternary complexes (rmsd = 0.306 Å). The protein electrostatic potential surfaces are shown separately for **b.** AtFLS2/flg22^{Pae}/AtBAK1 and **c.** GmFLS2b/flg22^{Rso}/GmBAK1.
PRR structures were obtained separately, using the Swiss-Model server, after previous sequence alignment with Blast, and restricting the target sequence to only the region represented in the pdb model of AtFLS2. The model structures were then structurally aligned with MUSTANG to the equilibrated triple complex structure of AtFLS2/AtBAK1+flg22^{Rso} (obtained as previously described) and further equilibrated by MD in explicit solvent, adopting the protocol described for the initial pdb structure.

Supplementary Figure 9

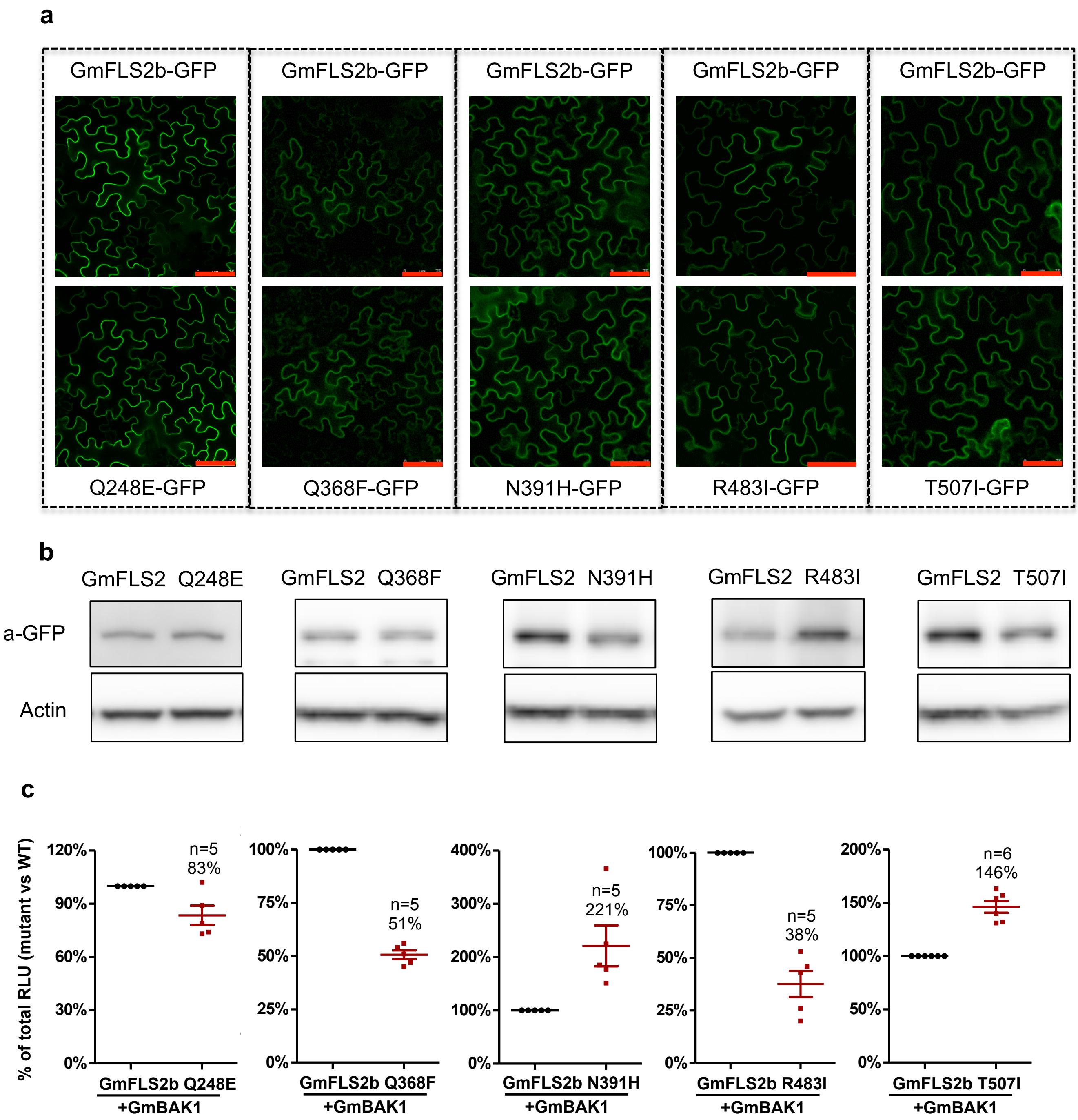

Supplementary Figure 9. Additional analyses of mutants in GmFLS2 residues important for perception of *R. solanacearum* flg22.

a. Subcellular localization of GmFLS2b-GFP and the indicated mutant versions in *N. bethamiana* leaf cells observed using confocal microscopy. Samples were taken from the leaves used in Figure 3j-n for ROS assays; mutant variants are shown next to their respective wild-type control expressed side-by-side in the same leaf. GFP fluorescence was visualized 2.5 days-post Agrobacterium infiltration. Scale bar=75 µm. **b.** Western blot to determine protein accumulation in the experiments shown in Figure 3 j-n, using an anti-GFP antibody. Molecular weight (kDa) marker bands are indicated for reference. An anti-actin antibody was used to show equal loading. **c.** Composite data of the different experiments performed to analyse responsiveness to flg22^{Rso} (100 nM) in *N. benthamiana* leaf tissues expressing GmFLS2b or the indicated mutant versions (with a C-terminal GFP tag) together with untagged GmBAK1. Representative results are shown in Figure 3j-n. Total ROS was represented as percentage (%) of the ROS accumulated in control samples (expressing wild-type GmFLS2b/GmBAK1) for each experiment; the % of each mutant for the different experiments (n is indicated in the figures) is represented in these graphs, together with the average value and the SEM.

Supplementary Figure 10

GmFLS2b-GFP	+	-	-	-	-	-	+	-	-	-	-	-
GmFLS2b-Q248E-GFP	-	+	-	-	-	-	-	+	-	-	-	-
GmFLS2b-Q368F-GFP	-	-	+	-	-	-	-	-	+	-	-	-
GmFLS2b-N391H-GFP	-	-	-	+	-	-	-	-	-	+	-	-
GmFLS2b-R483I-GFP	-	-	-	-	+	-	-	-	-	-	+	-
GmFLS2b-T507I-GFP	-	-	-	-	-	+	-	-	-	-	-	+
GmBAK1-HA	+	+	+	+	+	+	+	+	+	+	+	+
flg22^{Rso}	-	-	-	-	-	-	+	+	+	+	+	+

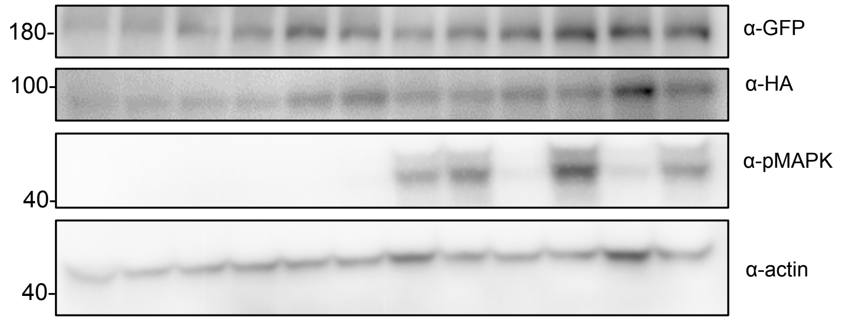

Supplementary Figure 10. MAPK activation phenotypes of mutants in GmFLS2 residues important for perception of *R. solanacearum* flg22.
GmFLS2-GFP or mutant versions were co-expressed with GmBAK1-HA in *N. benthamiana* leaves. Fourty-eight hours after Agrobacterium inoculation, 1µm flg22^{Rso} or water were infiltrated into *N. benthamiana* leaves, and samples were taken after 10 minutes. Western blots were performed using anti-phosphorylated MAPK antibody (anti-pMAPK). Immunoblots were also analysed using anti-GFP and anti-HA antibody to verify protein accumulation. Anti-actin was used to verify equal loading. Molecular weight (kDa) marker bands are indicated for reference.

Supplementary Figure 11

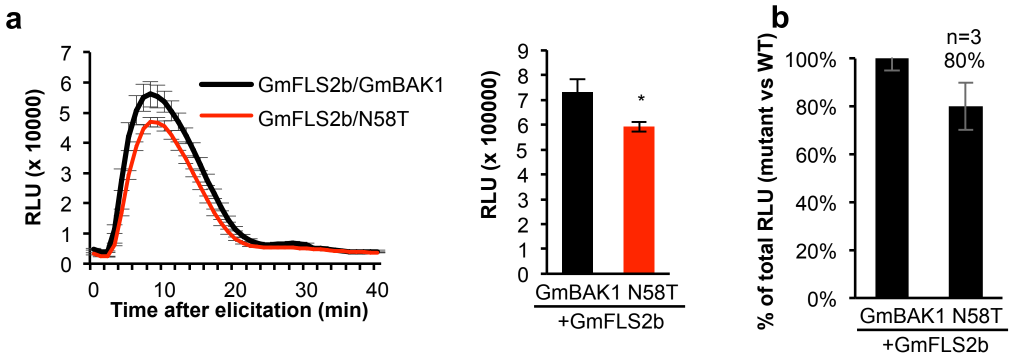

Supplementary Figure 11. Responsiveness of the GmFLS2/GmBAK1-N58T mutant complex to *R. solanacearum* flg22.

a. Dynamics of ROS burst triggered by flg22^{Rso} (100 nM) in *N. benthamiana* leaf tissues expressing GmBAK1 or GmBAK1-N58T (without tag) together GmFLS2b-GFP. ROS was measured in a luminol-based assay, and represented as accumulated relative luminescence units (RLU) (mean ± SEM, n=16). The measurement was performed from 1 to 40 min after treatment. Bar charts show accumulated relative luminescence units (RLU) from 5 to 40 min after treatment to avoid the effect of the background signal during the first 5 minutes. Asterisks indicate significant differences compared to the control expressing GmFLS2b and GmBAK1 as established by a Student's t test ($p<0.05$). **b.** Composite data of the different experiments performed to analyse responsiveness to flg22^{Rso} (100 nM) in *N. benthamiana* leaf tissues expressing GmFLS2b-GFP together with untagged GmBAK1 or GmBAK1-N58T. A representative result is shown in **a**. Total ROS was represented as percentage (%) of the ROS accumulated in control samples (expressing wild-type GmFLS2b/GmBAK1) for each experiment; the % of each mutant for the different experiments (n=3) is represented in these graphs, together with the average value and the SEM.

Supplementary Figure 12

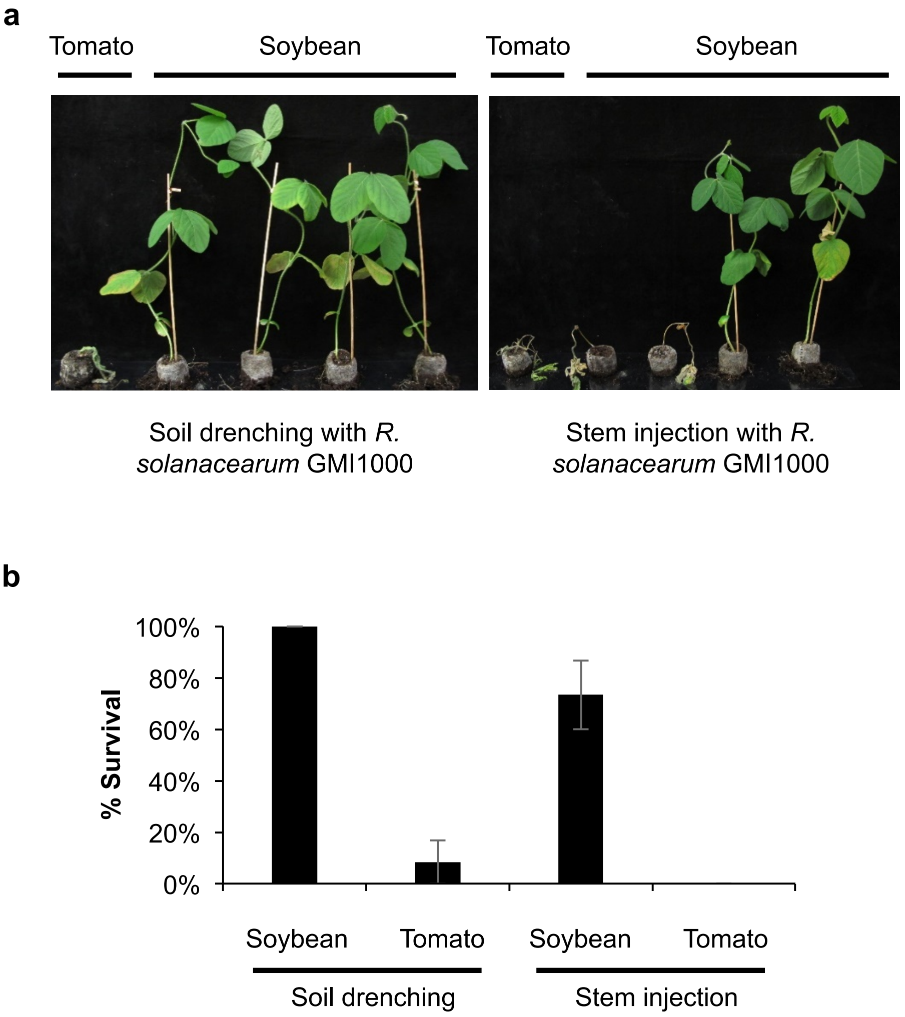

Supplementary Figure 12. Resistance of soybean to *R. solanacearum* GMI1000 in laboratory conditions.
a. Two week-old soybean or 4 week-old tomato plants were inoculated with *R. solanacearum* GMI1000 by soil-drenching (left) or stem injection (Right). Images were recorded 14 or 7 days after inoculation by soil-drenching or stem injection, respectively, and represent dead/alive plants as quantified in **b**. **b.** Percentage of surviving plants 14 or 7 days after inoculation by soil-drenching (n=12) or stem injection (n=10), respectively. These assays were performed 3 times with similar results.

Supplementary Figure 13

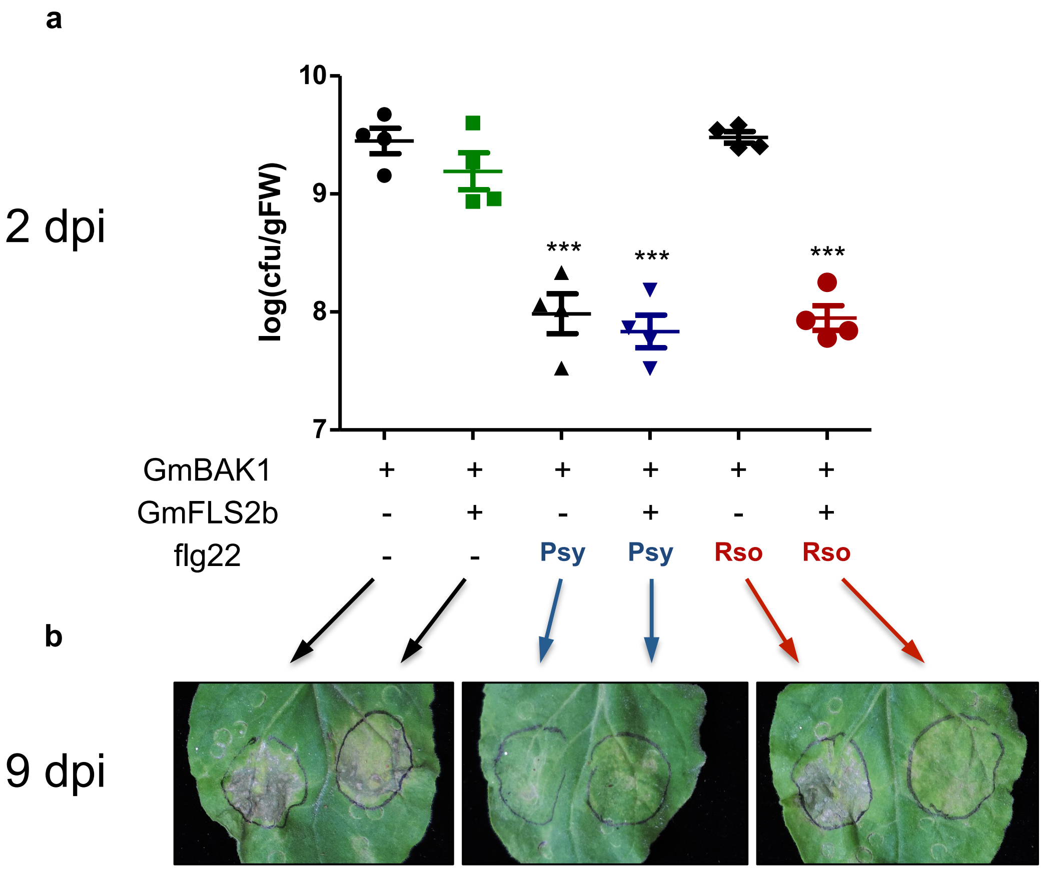

Supplementary Figure 13. Bacterial replication and symptom development upon *R. solanacearum* inoculation in *N. benthamiana* leaves.
a. Growth of *R. solanacearum* Y45 in *N. benthamiana* leaves expressing GmBAK1 (as control) or GmFLS2b/GmBAK1, as indicated. Leaves were pretreated with water (mock), 1 μM flg22^{Psy}, or 1 μM flg22^{Rso} for 12 h and then syringe-infiltrated with a 10^6 cfu/mL inoculum. Bacterial growth was determined 2 days post-inoculation (dpi) (mean ± SEM, n=4). Asterisks indicate significant differences compared to the mock control expressing GmBAK1 as established by a Student's t test (p<0.001). **b.** Macroscopic observation of disease symptoms in *N. benthamiana* leaf tissues treated as in **a**, recorded 9 dpi. These experiments were performed 3 times with similar results.

Supplementary Figure 14

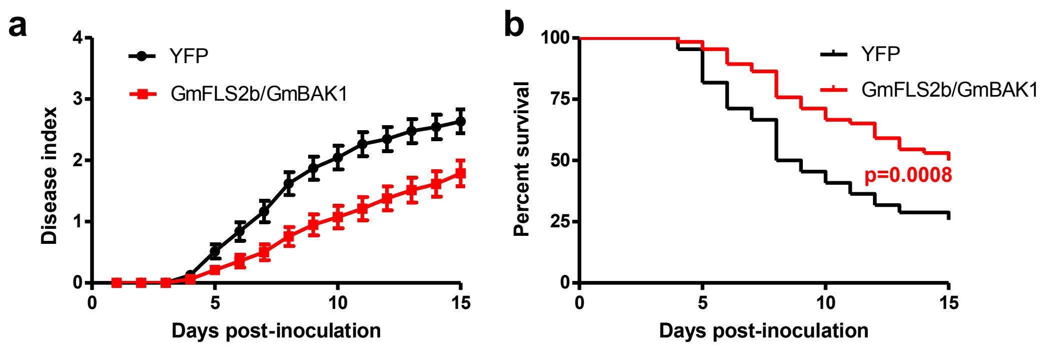

Supplementary Figure 14. Tomato plants with roots expressing GmFLS2b/GmBAK1 are more resistant to *R. solanacearum*.

a. Composite data from 4 independent replicates of the experiment shown in the figure 4c and d. Soil-drenching inoculation assays in tomato plants with roots expressing YFP (as control) or GmFLS2b/GmBAK1. Plants were inoculated with *R. solanacearum* GMI1000. The results are represented as disease progression, showing the average wilting symptoms in a scale from 0 to 4, and combining all the values from 4 independent experiments (mean ± SEM, n=66). **b.** Survival analysis of tomato plants in **a.** The disease scoring was transformed into binary data with the following criteria: a disease index lower than 2 was defined as '0', while a disease index equal or higher than 2 was defined as '1' for each specific time point. Statistical analysis was performed using a Log-rank (Mantel-Cox) test (n=66) to analyze the difference with the control, and the resulting p value is indicated in the figure.

Supplementary Figure 15

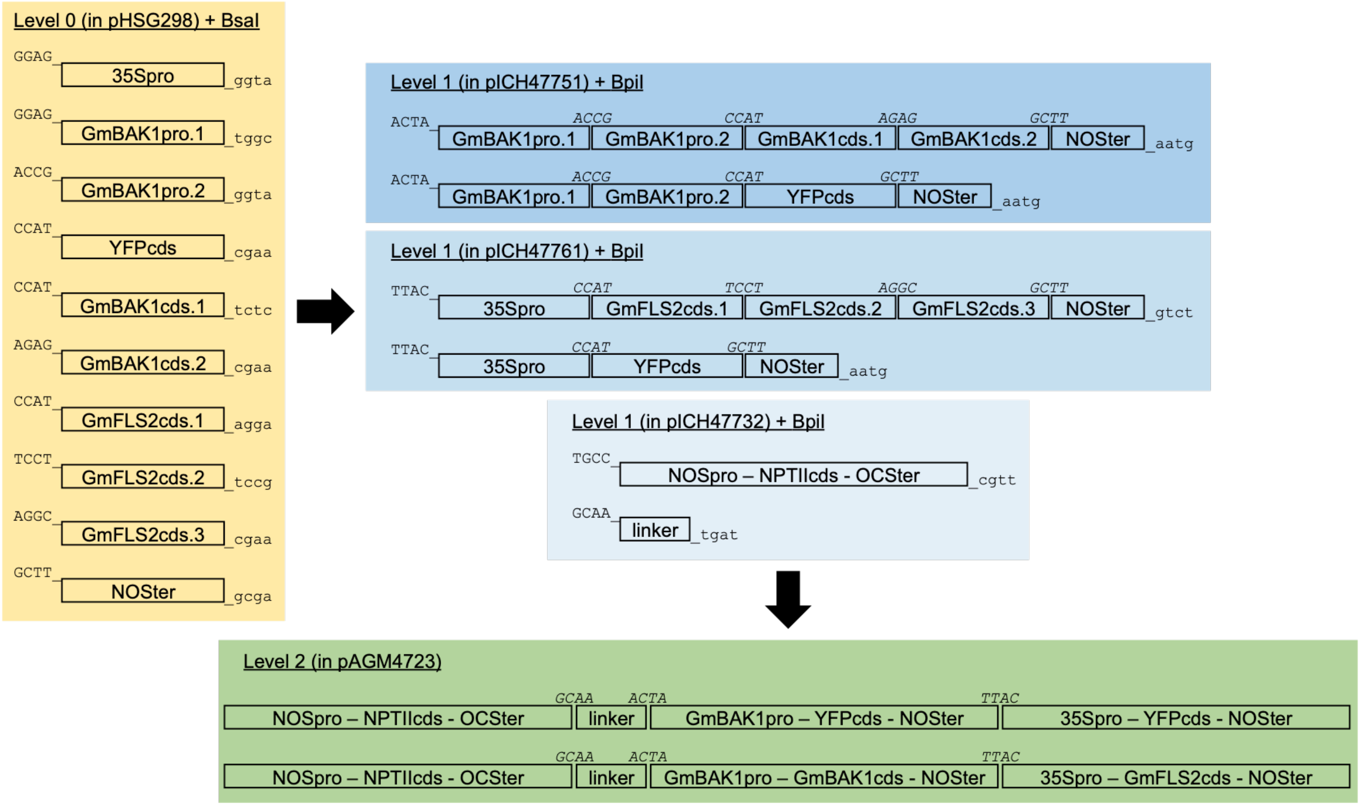

Supplementary Figure 15. Summary of the generation of the plasmid co-expressing *GmFLS2* and *GmBAK1* by Golden Gate technology.
A kanamycin resistance cassette (nopaline synthase (NOS) promoter, neomycin phosphotransferase (NPTII) coding sequence, octopine synthase (OCS) terminator), GmBAK1 or yellow fluorescent protein (YFP) coding sequence under the control of GmBAK1 promoter and GmFLS2 or YFP coding sequence under the control of the CaMV 35S promoter were assembled using MoClo technology (described by Engler et al., 2011) in the binary vector pAGM4723 (green). The level 0 modules and level 1 gene assemblies are shown in yellow and blue respectively. The 4 bp overhangs resulting from *Bsa*I or *Bpi*I restriction are shown in uppercase (5') and lowercase (3') and the ends of each fragment and on top of the ligated products. Pro: promoter; ter: terminator; cds: coding sequence.

Supplementary information

Supplementary Table 1. Primers used in this study

Gene	Forward primer	Reverse primer
**Cloning**		
AtFLS2	CACCATGAAGTTACTCTCAAAGACCTT	AACTTCTCGATCCTCGTTACGATCT
AtBAK1	GGGGACAAGTTTGTACAAAAAAGCAGGCTTCATGGAGAGAGTGACTTCATC	GGGGACCACTTTGTACAAGAAAGCTGGGTGTTATCTAGGACCTGATAGTTC
AtBAK1-no stop		GGGGACCACTTTGTACAAGAAAGCTGGGTGTCTTGGACCCGAGGGGTATTC
GmFLS2	GGGGACAAGTTTGTACAAAAAAGCAGGCTTCATGTTGTCCCTAAAGATTAGT	GGGGACCACTTTGTACAAGAAAGCTGGGTGACAAGACAGTGTTGTTTGAAGCTTC
GmBAK1	GGGGACAAGTTTGTACAAAAAAGCAGGCTTCATGGAGAGAGTGACTTCATC	GGGGACCACTTTGTACAAGAAAGCTGGGTGTTATCTAGGACCTGATAGTTC
GmBAK1-no stop		GGGGACCACTTTGTACAAGAAAGCTGGGTGTCTAGGACCTGATAGTTC
**Site-directed mutagenesis**		
Q248E	CAAATTTAGAATACCTTGAATTGTTCGAGAATTCATTGAGTGGGAAAGTTCCTTC	GAAGGAACTTTCCCACTCAATGAATTCTCGAACAATTCAAGGTATTCTAAATTTG
Q368F	CTTGACATATCTGTCAATGAGCTTCAATCTCCTCTCAGGTGAACTTC	GAAGTTCACCTGAGAGGAGATTGAAGCTCATTGACAGATATGTCAAG
N391H	ATGATTTGAAGTTTCTTGTTTTGCACTCCAACTGCTTTCATGGATC	GATCCATGAAAGCAGTTGGAGTGCAAAACAAGAAACTTCAAATCAT
R483I	CAGATATCCAGAATCTCTCTAAACTCATAATCCTGCAGCTGAATGG	CCATTCAGCTGCAGGATTATGAGTTTAGAGAGATTCTGGATATCTG
T507I	GGAAACTTGAATCAACTCGTCATTTTATCCCTTTCTGAAAATACG	CGTATTTTCAGAAAGGGATAAAATGACGAGTTGATTCAAGTTTCC
N58T	CGTGCAGGGAGTGACAAGGGTAGCATCCCA	TGGGATGCTACCCTTGTCACTCCCTGCACG

Supplementary Table 2. Peptides used in this study

flg22^{Rso}	QRLSTGLRVNSAQDDSAAYAAS
flg22^{Rso} -GLQIA	QRLSTGLRVNSAQDDSAGLQIA
flg22^{Rso} -GLQIS	QRLSTGLRVNSAQDDSAGLQIS
flg22^{Rso}-I-GLQIA	QRLSTGLRINSAQDDSAGLQIA
flg22^{Psy}	TRLSSGLKINSAKDDAAGLQIA
flg22^{Psy} -I21A	TRLSSGLKINSAKDDAAGLQAA
